# The Registration of Medical Students

**Published:** 1904-06-18

**Authors:** 


### The Hospital.

A JOURNAL OF

The Medical Sciences and Hospital Administration.

VOL. XXXVI.—No. 925. JUNE 18, 1904.

### THE REGISTRATION OF MEDICAL STUDENTS.

THE long dispute between the General Medical Council and the Royal Colleges of Physicians of London and of Surgeons of England seems at last likely to be terminated by what is practically an intervention of the Privy Council in favour of the first-named body, and in favour, it will generally be thought, of the interests of the public in effective medical education. It has been becoming yearly more evident, to most of those who have been called upon to consider the matter, that effectiveness in this respect is largely a matter of foundation, and that, without some real knowledge of the elements of organic chemistry, of physics, and of biology, the seed sown by special medical teaching is likely to fall upon comparatively unfertile ground. The Medical Council, by proceedings which from time to time we have brought under the notice of our readers, has endeavoured to promote the necessary improvements in preliminary education; but the colleges, rather, we believe, from a desire to assert their own privilege of initiative than from any other reason, have resisted the efforts of the Council, and have continued to accept the so-called scientific teaching of common boys' schools as an essential part of the medical curriculum, and as proper to be included in an estimate of its duration. The Council met this opposition by laying down conditions to be fulfilled as preliminaries to registration by medical students; and the colleges replied that the Council had no right to demand registration, which was merely an arrangement agreed to by all parties for the sake of mutual convenience, and with which they, for the future, would dispense. They therefore ceased to require registration from students who intended to seek their respective licenses or diplomas, and were for a time masters of the situation; and from this point of vantage they expressed an intention of really considering the merits of the case, and of becoming more exacting in their "preliminary" requirements. At the last session of the Council, however, a proposal, which it was an open secret had been suggested by the Privy Council, was carried, to the effect that the Government should be requested to introduce a Bill into Parliament by which the Medical Council would obtain statutory power to require registration as a preliminary to the commencement of medical study, and to lay down the terms under which such registration should be effected. In the present state of political parties and of public business it is impossible to be very sanguine concerning the prospects of any projected measure, however good or useful; but the suggestion may in the meanwhile be welcomed, as showing that the Government is not entirely indifferent to medical questions, or unconscious of the bearing which they may have upon the welfare and safety of the public. If it should ever be carried into practical effect the authority of the Council over preliminary education will be rendered absolute, and the questions which have been so warmly debated during the last few years will be settled in the interests of efficiency alone. Legislation of this kind might easily be carried a step further, and might be made to simplify the present complicated condition of the subsequent or purely professional part of the curriculum and of the examinations. The licensing corporations would do wisely to reflect upon the weighty words in which the President of the Council, Sir William Turner, pointed out that the language of old charters could not be suffered perpetually to stand in the way of changes which had been rendered necessary by a changed order of events, and that even the holders of such charters must be prepared to accommodate themselves to necessities. There is a very general feeling that some of the nineteen existing doors of entrance to the medical profession might be closed with great advantage and that it would be much more easy to exert an effective control over a single qualifying examination, or over a single one for each division of the kingdom, than over the present almost bewildering variety of boards, of licenses, and of corporations. The proposal to give the Medical Council uncontrolled authority in one department of its work may, therefore, bear a suspicious resemblance to our old friend, "the thin end of the wedge." The Council has given abundant evidence of the care and discretion with which its hitherto limited powers have been exerted, and, having thus been faithful over a few things, it may possibly look forward to being trusted with many.

In view of this prospect, the proposed Bill contains another provision of no small importance, a proposal that the Council should not only be empowered to keep a register of all medical and dental students, but also that the students in question should pay a fee, not exceeding a pound, for the privilege of placing their names upon it, and of acquiring thereby a definite status among the gentlemen who were described by Sam Weller as "Sawbones in training." It is calculated that the fee in question would add about £1,700 a year to the pecuniary resources of the Council; and, as that body has for some years been spending from its accumulations, and in excess of its income, to the extent of about £1,500 a year, the increase of revenue would not only be extremely welcome, but would speedily become necessary if the functions of the Council are still to be discharged. For some years past, indeed, the consideration of the annual financial statement has been productive of what has been called the ignoble form of melancholy which attends upon pecuniary embarrassment, and various devices for the curtailment of expenditure have from time to time been considered and abandoned. More than one member of the Council has suggested an abandonment of the fees now received by members, but the general opinion has always been, and, as we conceive, rightly, that the labourer is worthy of his hire, and that the business of the State ought not to be transacted gratuitously. The most practical suggestions for reform were that no one but the chairman of a committee, in introducing a report, should speak for more than ten minutes at a time, and that no "direct representative" should speak more than twenty times in one debate. But the Council, on the whole, is a conservative body, in which such sweeping changes would be regarded with disfavour.

